# Recruitment of Youth Living With HIV to Optimize Adherence and Virologic Suppression: Testing the Design of Technology-Based Community Health Nursing to Improve Antiretroviral Therapy (ART) Clinical Trials

**DOI:** 10.2196/23480

**Published:** 2020-12-11

**Authors:** Allison Lorna Agwu, Hasiya Eihuri Yusuf, Lawrence D'Angelo, Mobeen Rathore, Jeanette Marchesi, Julia Rowell, Raina Smith, Jackie Toppins, Constance Trexler, Rashida Carr, Betty Johnson, Aaron Keith Selden, Saniyyah Mahmoudi, Susan Black, Jisell Guadamuz, Steven Huettner, Maria Trent

**Affiliations:** 1 Department of Pediatric and Adult Infectious Diseases Johns Hopkins School of Medicine Baltimore, MD United States; 2 Johns Hopkins School of Medicine Baltimore, MD United States; 3 Children's National Medical Center Washington, DC United States; 4 University of Florida Center for HIV/AIDS Research, Education and Service University of Florida College of Medicine Jacksonville, FL United States; 5 University of Florida College of Medicine Jacksonville, FL United States; 6 Johns Hopkins Bloomberg School of Public Health Baltimore, MD United States

**Keywords:** adolescent, youth, community health nursing, HIV, adherence, viral suppression, mobile health

## Abstract

**Background:**

Despite advances in HIV diagnosis and treatment, adolescents and young adults 12-25 years old have high HIV incidence, poor engagement and retention in treatment, and low rates of adherence and virologic suppression when compared to their older counterparts. HIV has emerged as a chronic disease for which antiretroviral therapy (ART) adherence is critical for virologic suppression and long-term survival. Virologic suppression has been elusive for many youth with HIV (YHIV). Novel strategies designed to facilitate health care systems’ support for YHIV between medical visits are essential for improving ART adherence, virologic suppression, and long-term survival.

**Objective:**

The aim of this study is to compare the effectiveness of a technology-enhanced community health nursing intervention (TECH2CHECK) to a standard of care (SOC) control group for improving ART adherence and subsequent viral suppression using a randomized trial design. The objectives are to assess the feasibility, acceptability, and cost-effectiveness of TECH2CHECK as compared to SOC for management of HIV in the outpatient setting and to examine the sustainability of self-care behavior, adherence, and virologic suppression among youth following the intervention period.

**Methods:**

We will recruit 120 adherence-challenged YHIV being followed at clinics specializing in HIV care in the Baltimore-Washington metropolitan area and in Jacksonville. Eligible participants complete an audio, computer-assisted self-interview and are randomized to either TECH2CHECK intervention or the SOC (60 participants in each arm). The primary outcome of interest is virologic suppression (viral load <20 copies/mL) and improved treatment adherence. Participants in the intervention arm receive community health nursing visits at 2 weeks, 6 weeks, 10 weeks, 14 weeks, and 26 weeks. The intervention arm also receives SMS messaging comprising daily adherence and appointment reminders and positive reinforcement for medication adherence daily for 2 weeks, on alternate days for 2 weeks, thrice weekly for 1 month, weekly for 3 months, and every 2 weeks for the rest of the study duration. The control group receives appointment reminders and SOC per clinic protocol. Exploratory analysis will be conducted to determine differences in medication adherence and virologic suppression in the 2 arms and to assess cost-effectiveness and study feasibility and acceptability.

**Results:**

In the first 23 months of the study (July 2018-April 2020), 56 (55%) of 102 eligible patients were enrolled and randomized. At present, participating youths are primarily African American (53/56, 95%), male (37/56, 66%), and ≥18 years old (53/56, 95%). Follow-up study visits, as required per the protocol, have been completed by 77% (43/56), 94% (45/48), 95% (37/39), 96% (24/25), and 100% (10/10) of participants at the 1-month, 3-month, 6-month, 12-month, and 18-month follow-ups, respectively.

**Conclusions:**

Preliminary accrual and retention data suggest that TECH2CHECK is feasible and acceptable.

**Trial Registration:**

ClinicalTrials.gov NCT03600103 https://clinicaltrials.gov/ct2/show/NCT03600103

**International Registered Report Identifier (IRRID):**

DERR1-10.2196/23480

## Introduction

### Background

Youth aged 12-24 years comprise 21% of all new HIV infections in the United States, with 8000 diagnoses yearly [1]. In this age group, the high infection rate is coupled with poor engagement and retention in care, low rates of treatment initiation, and low rates of antiretroviral therapy (ART) adherence and virologic suppression [2,3]. The HIV care continuum is a framework for understanding the status of persons living with HIV (PLWH), including being aware of HIV diagnosis, linkage to care, retention in care, receipt of ART, and attainment and maintenance of virologic suppression [4]. It is estimated that only 6% of youth with HIV (YHIV) are virologically suppressed [5]. Failure to initiate treatment and attain suppression increases the risk of disease progression and transmission in sexual networks [6]. Adherence to outpatient provider visits, filling prescriptions, and consistent use of ART are the keys to virologic suppression. However, durable virologic suppression eludes many YHIV due to a combination of cognitive, developmental, and psychosocial challenges specific to this population. Barriers to care include stigma and lack of accessible and affordable youth-friendly services along with poor health literacy [7,8]. Even in adolescent-focused clinics like the 13 clinics that comprise the National Institutes of Health–funded Adolescent Medicine Trials Network, youth continue to have challenges. Recently published data on 1411 youth aged 12-24 years who were referred to one of the Adolescent Medicine Trials’ Network for HIV/AIDS sites through SMILE (Strategic Multisite Initiative for the Identification, Linkage, and Engagement in Care of Youth) demonstrated that, even with dedicated outreach workers and tailored initiatives, only 12% of youth achieved viral suppression after a median of 5 months [9]. Youth with poor virologic control are predisposed to antiretroviral drug resistance, immunologic decline, and greater health care costs (hospitalizations, emergency department utilization) [10-12]. When given the tools, YHIV are able to engage and adhere to ART, though there are limited evidence-based approaches specifically targeted to this population [13]. Short-term interventions (eg, directly observed therapy, financial incentives) have had limited success and sustainability [13]. Given the consequences and costs of nonadherence to care and ART in this population, novel strategies that remove barriers to care and allow for provision of care for YHIV between medical visits and outside of the clinic setting are essential to improving outcomes for YHIV. Rigorously designed and tested strategies using randomized controlled designs are therefore critical to reducing the treatment disparities observed among YHIV.

Efforts to improve the care continuum among YHIV have generally focused on the first 2 components of diagnosis and linkage to care. However, modeling by Shah et al [14] indicates that smaller population-level impact (reduced incidence, survival) is seen with improvements in the upstream components of the care cascade (diagnosis, linkage), while more dramatic effects occur with improvement in the latter components (retention, sustained virologic suppression) of the care continuum [15,16].

With the advent of ART, HIV has evolved into a chronic disease with life expectancy equivalent to that of uninfected individuals [17,18]. The current model of care includes patients attending appointments with their HIV provider quarterly or less frequently. The reliance on patient responsibility for follow-up in care may pose challenges for YHIV. The Department of Health and Human Services HIV guidelines state that PLWH can be seen by their provider as infrequently as once yearly if stable [19]. Our data have demonstrated that reduced frequency of HIV provider visits (<3 in a calendar year) is indeed associated with decreased rates of ART initiation and continuation among YHIV [20,21]. Importantly, this profile is associated with higher hospitalization rates due to HIV-related and unrelated diagnoses for YHIV [11]. Effective HIV management of YHIV may require a model of care different than what is currently being employed. Alternate models of care are being explored for management of individuals with chronic illnesses, particularly for youth, where flexibility, convenience, and technology may be invaluable components of their care [22]. We will therefore need to consider interventions that change the paradigm (eg, enhance delivery systems, strengthen the health system, improve self-management support, inform and activate the patient) and thereby sustainably improve outcomes.

Evidence-based interventions that specifically target YHIV are understudied. A systematic review of adherence interventions targeting YHIV 13-24 years old reported on outcomes of adherence, viral load (VL) suppression, or CD4 from 10 studies (8/10 US-based) [23]. The review included 346 YHIV with an average of 35 participants per study. The varied interventions included directly observed therapy, cell phone and text message reminders, guided problem solving about adherence, motivational interviewing, other reminder strategies, individual and family counseling, and financial incentives, lasting from 12 weeks to 24 weeks, with outcomes assessed at 24-96 weeks. The studies were limited, as most were pilot studies without a control group, and underpowered to detect statistical differences. Two randomized controlled trials (RCTs) that utilized individualized cell phone adherence reminders with problem-solving strategies and weekly in-person or internet-facilitated patient and family counseling for training and skills building resulted in improved adherence, as confirmed with reduced VL measures after 12-14 months [24,25].

When considering potential interventions, the feasibility of delivery mechanisms is critically important, with much attention paid to technology integration. A 2018 nationally representative survey of adolescents found that 95% owned or had access to a smartphone [26]. African American youth were most likely (85%) to have smart phones. US cell phone penetration currently stands at 120.7%, up from 69% in 2005 [27], and there is high penetration of cell phones or smart phones among YHIV cared for in the participating clinics [28]. Text messaging is a viable method of successfully communicating health messages with patients, including a successful sexual health text messaging service (SEXINFO), which improved basic sexually transmitted infection and HIV information and referral sources for in-person consultation for San Francisco youth [29]. It has also been shown to improve virologic suppression and retention in care in HIV-positive youth on ART and in promoting sexual health education among sexually active HIV-negative youth in the United States [30,31]. Text messaging has become an essential component of daily life for YHIV that needs to be optimized from a systems perspective to extend care and adherence support [32].

Community health nurse (CHN) home visiting interventions have been shown to reduce repeat pregnancy rates [33], improve utilization of resources for pregnant and parenting adolescents [34], and optimize care for chronic diseases like asthma [35]. The maximum benefit is seen with participants at highest risk when nurses are assigned solely to implement a protocol [36]. The TECH-N study, a community-based nursing intervention study, also demonstrated that delivery of CHN services in the community was more successful than referral for clinic-based services and proved to be highly feasible and acceptable among low-income youth living in neighborhoods with high sexually transmitted infection or HIV prevalence who were diagnosed with acute pelvic inflammatory disease [37]. The CHN model has not been specifically studied among YHIV 12-25 years old [38]. One Ugandan study examined the benefits of long-term (average 4.2 years) community health–based care vs a clinic-based approach in children 0-18 years of age living with HIV, demonstrating better retention, but no difference in survival [39]. The TECH2CHECK intervention was designed to address unmet care needs designed to further assist YHIV to reach undetectable status. The aim of this trial is to specifically evaluate the effectiveness of the intervention on retention in care, ART initiation and maintenance, and rates of virologic suppression among urban minority YHIV who have been unable to reach virologic suppression. The cost-effectiveness of the intervention will also be assessed.

### Objectives

The primary aim of the TECH2CHECK study is to compare the effectiveness of TECH2CHECK to standard of care (SOC) in improving viral suppression using an RCT among urban YHIV demonstrating suboptimal adherence to treatment as evidenced by HIV viremia (VL >20 copies/mL). We will compare the feasibility, acceptability, and cost-effectiveness of TECH2CHECK to SOC for management of HIV in the outpatient setting and examine the sustainability of self-care behavior, adherence, and virologic suppression among youth following the intervention period. Drawing from the success of the TECH-N study, we will extend the same skills to study the challenges among YHIV [37]. Given the convincing TECH-N data and the similarities between YHIV targeted in this project, we repurposed the CHN approach used in TECH-N for nonadherent YHIV. The aim of this paper is to describe the methods and preliminary recruitment, intervention delivery, and retention outcomes of TECH2CHECK as an initial assessment of the feasibility of this work.

## Methods

### Study Design, Population, Sampling, and Randomization

The study is a single-blinded RCT enrolling YHIV in Baltimore, Maryland (site 1), Washington, D.C. (site 2), and Jacksonville, Florida (site 3). The Baltimore recruitment site is made up of 2 interconnected clinics, both geographically located in one of the highest HIV prevalence areas in the city. All 3 clinical sites are referral sites for newly diagnosed youth, actively involved in HIV clinical service delivery using national standards and proactively involved in youth-centric HIV-related research activities. The most recent data from the study-affiliated clinics reveal high viremia rates (VL >20 copies/mL) among 12-25-year-olds of 30-36% (Site 1), 34% (Site 2), and 60% (Site 3). All the clinics are located in areas targeted in the government’s Ending the HIV Epidemic: A Plan for America [40].

**Figure 1:**
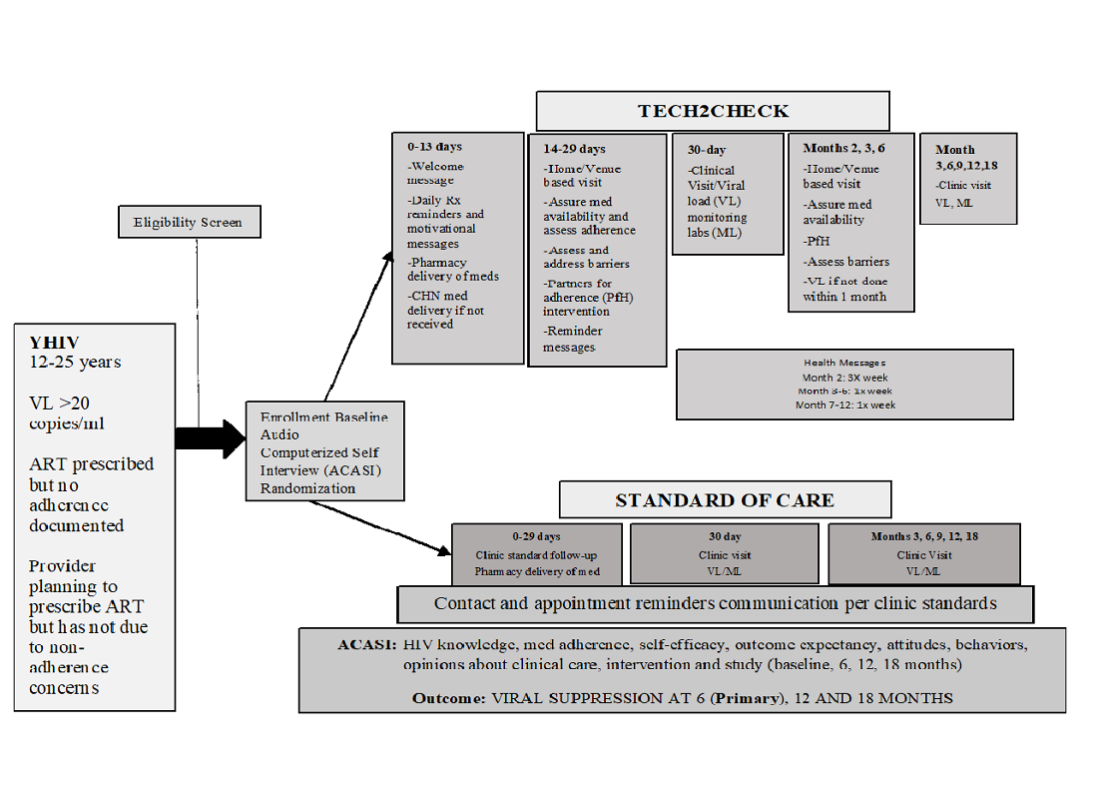
Schematic of TECH2CHECK design. ART: antiretroviral therapy; CHN: community health nurse; YHIV: youth with HIV.

We are enrolling and randomizing 120 YHIV aged 12-25 years with HIV nonadherence to either TECH2CHECK (Arm 1) or SOC control group (Arm 2) using a permutated block design to ensure group balance at each of the sites at the end of the trial period. This strategy helps to minimize any systematic differences between patients enrolling at different times during the study. Participants in Arm 1 (intervention group) receive community-based health visits, SMS text messages with positive health messages, ART and clinic appointment adherence reminders, and a 3-5-minute behavioral intervention described in a later section. In Arm 2 (control group), YHIV receive SOC as dictated by their primary HIV provider and clinic standards and communication and appointment reminders as per the standard clinic practice. Participants in both arms have their regularly scheduled clinic visits as dictated by their provider (usually every 3 months; see Figure 1). The protocol is described according to the CONSORT-EHEALTH checklist [41].

### Recruitment, Eligibility, and Informed Consent

YHIV are being recruited from the clinical sites by trained research staff after being referred by providers and clinical teams during routine clinical care visits for this study approved for human subjects. Eligible participants are aged 12-25 years, have been diagnosed with HIV, are prescribed ART or eligible for ART but nonadherent to initiation, have VL >20 copies/mL, permanently reside in the recruitment area, and are willing to sign informed consent. Informed consent includes willingness to complete study procedures, agreement to study randomization and community-based follow-up by our team, and the ability to speak and read English. Patients are excluded if there is existing mental health, cognitive, or behavioral dysfunction that would impair effective participation if present or in the event of severe illness requiring hospitalization at the time of enrollment. Consent will be obtained directly from subjects aged ≥18 years and from parents or guardians of younger subject across all but one site where parental consent is not required for the enrollment of younger patients.

### Training, Follow-Up, and Tracking

Research staff have been trained on the recruitment and consent protocol, interview surveys, and confidentiality of data collection. Ongoing communication between the sites enables the team to identify and address barriers to referral or recruitment. Involvement of key study personnel in clinic leadership further enhances the focus on the study. Research staff track eligible patients, those approached, and outcome of the approach. Detailed contact information obtained from the patient at the time of the initial recruitment includes home and work numbers, cell phone numbers, email addresses, Facebook, other social networking sites (user names and IDs), school attended, and the names of 2 individuals who usually know their whereabouts and can be contacted to locate them. Participants are advised to notify study staff of changes in their living environment or contact info and receive a small US $5 gift card incentive for this notification, a successful initiative from the TECH-N trial [37].

### Intervention and Control Conditions

TECH2CHECK involves field visits by a CHN trained in disease intervention protocols, including clinical assessment, case management, counseling, and behavioral intervention. The CHN conducts community-based visits at mutually agreed-upon home and venue-based locations monthly or every 3 months. After enrollment, the first CHN visit occurs within 1-3 weeks of the enrollment or randomization, then at 2 weeks, 6 weeks, 10 weeks, 14 weeks, and 26 weeks. Nursing visits stop at 6 months, but participants and nursing staff will maintain communication via phone and the Emocha messaging app [42].Visits include clinical assessments, laboratory draw and assessment, adherence counseling and management, symptom assessment, and case management. CHNs assess ART adherence and actively assist with ART delivery as needed. At the end of the visit, the CHN determines a plan of action using 2 conditional algorithms based on the clinical assessment: one for participants requiring immediate attention (eg, symptoms needing acute management) and the other for participants for whom nonimmediate follow-up is indicated (eg, patient has nonurgent medical or psychosocial needs). The CHN nurse gives referral resources to assist patients who may have other issues identified during the visit (eg, utilities, housing). SMS messages comprising adherence reminders, positive reinforcement for HIV management, and appointment reminders are provided to participants in the intervention arm. Specifically, they receive a welcome message, daily SMS messages, and directly observed treatment in the first month; SMS messages thrice weekly in the 2nd month; SMS messages once weekly for months 3-6; and weekly reinforcement and retention messages for months 7-18. Communication via study cell phone allows for 2-way communication so that participants can both respond and initiate. Integral to the TECH2CHECK intervention is the use of an effective behavioral intervention to improve HIV adherence and self-care behaviors. TECH2CHECK utilizes the Centers for Disease Control and Prevention’s Partnership for Health to provide skill-based adherence and risk-reduction counseling [43]. Standard operating procedures were developed for triage and referral if the CHN goes out and the participant has an acute issue or complaint. The CHNs have 24/7 back up by the site principal investigators to assure clinical coverage by a physician.

In the control arm, patients receive SOC and appointment reminders. After completion of study enrollment procedures; an audio, computer-assisted self-interview (ACASI) baseline survey; and collection of baseline blood draw, the usual SOC as dictated by the provider is employed. The participant is given instructions for medication administration and information about side effects, and their next appointment is scheduled prior to leaving the clinic. All communications and interactions are performed per the clinic standard and recorded by the study team.

### Data Collection and Measures

The primary outcome measures of virologic suppression and secondary outcome measures of clinical outcomes (pharmacy refill, medication adherence, completed encounters, visits) and cost-effectiveness will be assessed at the end of the study. We will build upon and adapt the previously developed ACASI survey to collect perceived barriers to treatment and self-efficacy data from YHIV. The measures on the baseline survey include demographics, HIV treatment and adherence history, disclosure, mental health, substance use, sexual history, HIV adherence self-efficacy and perceived barrier scales, social provisions scale, and the short-form survey instrument (SF-12) as a measure of health-related quality of life [44]. Medical history (clinic attendance, hospitalization, acute care visits), medication adherence (self-reported adherence, pill counts, pharmacy records), side effects, non-ART medication usage, vital signs, symptoms, and visit diagnoses are obtained at all visits. At the TECH2CHECK nurse visits, the CHN records contact tracing data and performs a detailed clinical assessment including interval medical history, symptom reporting, medication usage, supportive care, side effects, activity level, and patient education. Permission to obtain medical records and health providers, if health care utilization is reported, is requested at the time of enrollment so that project staff can verify self-reported clinical data in the case of unreturned forms.

Standard HIV measurements include CD4, VL, and toxicity monitoring (chemistries) collected at clinic visits. VL and chemistries are collected by CHN at home or venue-based visits in the intervention arm only if not collected within the prior 4 weeks. Samples for CD4 measurements are collected by the CHN at 26 weeks if missed in the past 4 months.

### Feasibility and Acceptability

At the end of the study, data on study feasibility, acceptability, and refusal rates will be reported. Follow-up completion and study dropout rates will be used as indicators of study acceptability. Collection of basic demographic data and the outcome of recruitment effort will be conducted to determine the number of referral patients who are ineligible or decline to participate in the intervention and the similarities and differences from those who ultimately participate.

### Study Incentives and Benefits

All participants receive a US $25 gift card at enrollment and US $35 for each completed ACASI (baseline, 3 months, 6 months, 12 months, and 18 months). YHIV in the TECH2CHECK intervention arm do not receive compensation for home visits or for clinic visits attended given the current standards of care and because adherence to the CHN clinical visit is a behavior under study that must be made without any form of additional incentive. Transportation fare is provided to participants in both arms for SOC care visits and for labs as needed. We provide a disposable cell phone to allow for SMS messaging to those who are assigned to the intervention arm but do not have a cell phone. Overall, we anticipate this to be a minority of the youth given the 93% cell phone penetration among US youth, which we have confirmed in our prior local studies [26].

### Sample Size and Power

From the 4 participating clinics, we expect to enroll 144 nonadherent YHIV subjects over the period of this study, with about 120 subjects agreeing to participate and be evaluable for the primary outcome at 6 months and 12 months. Attrition rates of 10%-30% over the course of the study indicate that the study will have 80% (α=.05) power to detect differences in the proportions with virologic suppression between the control and intervention groups of at least 30%, assuming that the control group’s rate is 30%-40%.

### Data Analysis

The overall approach will be an intention-to-treat analysis. To test the hypotheses, in addition to data cleaning and exploratory data analysis, we will first review the demographic characteristics of the 2 study groups and between the clinical sites. Additionally, we will explore the characteristics of survey nonresponse and attrition. The plan for addressing each of the challenges and for hypothesis testing is described below. Data analysis will be performed using STATA Version 15.0 or comparable statistical software and the Tree Age software. The extent of data missing due to survey nonresponse will be assessed; two approaches, multiple imputation and pattern-mixture-modeling, will be considered for adjusting [45]. We present a descriptive analysis of the preliminary recruitment, baseline demographics, intervention delivery, and retention to assess feasibility and acceptability.

## Results

**Figure 2:**
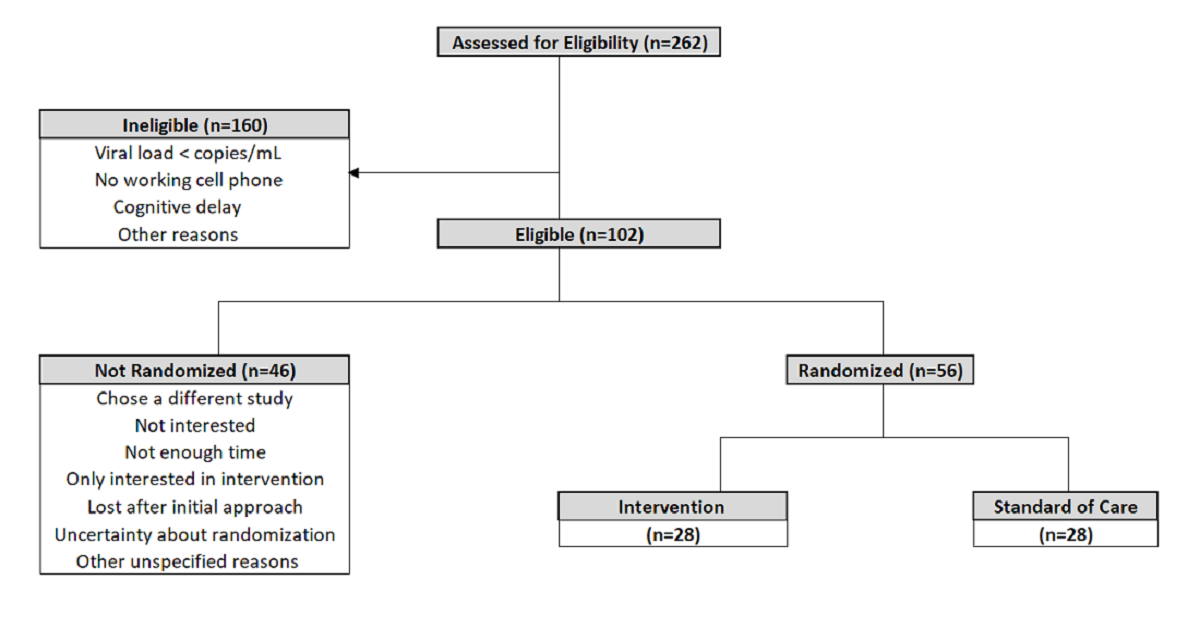
Recruitment of Study Participants.

The study opened for accrual in July 2018 at the Baltimore site, followed by the D.C. site in Jan 2019, and is currently ongoing. Due to unforeseen changes in the research staff infrastructure, the D.C. site ceased enrollment in June 2019 but continues to follow participants that were previously enrolled in the study. The team subsequently recruited and implemented the study at an additional site in Jacksonville, Florida; they began enrollment in February 2020. As of April 2, 2020, 262 patients were assessed for eligibility, and 38.9% (102/262) were deemed eligible and approached; 56 (56/102, 54.9%) were successfully recruited into the study (Figure 2). Of these, 95% (53/56) are African American, 66% (37/56) are male, and 95% (53/56) are ≥18 years old. Median VL at enrollment was 90 copies/mL (IQR 1553 copies/mL). Study accrual is on track and expected to be completed as planned. Participants are spread across different follow-up time points, reflecting the variations in the time of recruitment into the study. Presently, 77% (43/56), 94% (45/48), 95% (37/39), 96% (24/25), and 100% (10/10) of participants at the 1-month, 3-month, 6-month, 12-month, and 18-month follow-ups, respectively, have completed follow-up study visits as required per protocol. Additionally, 77% (20/26), 78% (21/27), 81% (21/26), 96% (23/24), and 90% (18/20) of subjects expected to receive CHN visits at 2 weeks, 6 weeks, 10 weeks, 14 weeks, and 26 weeks, respectively, have completed those visits. Of the 56 enrolled participants, 98% (55/56) have completed some or all study follow-ups; only 1 patient was lost to follow-up. There have been no safety concerns nor significant adverse events.

Final data collection and conclusion of the study are anticipated for July 2022, although research disruption due to the COVID-19 pandemic may extend the study completion timeline [46]. Formal analysis of data to evaluate the primary aims will proceed following study completion of data collection.

## Discussion

Findings from this study show that the recruitment of YHIV with poor medication adherence and virologic suppression is both feasible and acceptable. Virologic suppression among YHIV on ART remains suboptimal when compared with older age groups, as less than 30% of YHIV on ART achieve virologic suppression compared to 50% of adults [44,47]. The need for ART treatment delivery beyond the traditional clinic-based model and strategies to optimize treatment adherence in YHIV is highlighted by a growing body of evidence on the comparatively low adherence rates observed in youth in care for HIV [9]. TECH2CHECK builds upon past advancements in YHIV and addresses existing gaps in youth virologic control. First, we focus on identifying the impact of a community health intervention on adherence to ART by YHIV. While there have been a few RCTs that have sought to improve ART adherence through community-based interventions, the outcomes assessed are limited by the nonobjective assessment of adherence (ie, no viral load measurements) [48,49]. The statistical power of our study allows for the determination of statistically significant differences in the efficacy and cost-effectiveness of the intervention between both arms, if present, a frequently missing piece from studies [50,51]. An extended follow-up period of 18 months as opposed to shorter durations of follow-up will also allow for the assessment of long-term adherence, virologic suppression, and associated positive behavioral changes related to the intervention [48,50,51]. As there is a need for real-world interventions that address diverse YHIV infection with poor ART adherence whose behaviors and attitudes may differ by acquisition category, TECH2CHECK will include both youth who have acquired HIV perinatally (~30% of YHIV) and through high-risk behaviors [50,52,53]. Finally, we have chosen a viral load of >20 copies/mL as an indicator of nonadherence because, with the potency of newer regimens, we are of the opinion that elevations in VL even at these low levels often indicate adherence challenges and thereby allow deployment of adherence interventions before behavior patterns become solidified and before complete loss of virologic control [52]. Given the paucity of data on the effectiveness of alternative methods for improving ART adherence among YHIV, we anticipate that TECH2CHECK will be cost-effective and efficient in addressing barriers to ART adherence, improving retention in care and thereby leading to an improvement in the elusive control of HIV among YHIV, with potential for a substantial health impact. Although studies integrating community health interventions have been used in the TECH-N study of youth diagnosed with pelvic inflammatory disease, TECH2CHECK represents the first of its kind among YHIV in the United States and can potentially improve the status quo of YHIV [37].

### Limitations

An important limitation to consider is that the intervention may not be generalizable to clinics and locales serving nonminority populations. However, given that the demographics of YHIV in the United States resemble that of those that we anticipate will be enrolled in TECH2CHECK, this is less of a limitation. If effective, the intervention could subsequently be modified for other populations. Additionally, the current COVID-19 pandemic and shelter in place orders have impacted some aspects of the study as recruitment has been temporarily halted. However, all study sites continue to work remotely, collection of study data has continued, and nursing visits have been replaced by virtual visits. While it is difficult to estimate the impact of the COVID-19 pandemic on enrolled participant behavior within the study at this time, we recognize how vitally important it is to assess this in the final analysis at the end of the study. We have had extensive experience recruiting youth at risk for and living with HIV through our activities in the TECH-N study, Adolescent Medicine Trials’ Network for HIV/AIDS, and collaborations with our Centers for AIDS Research, International Maternal Pediatric Adolescent AIDS Clinical Trials, and HIV Prevention Trials’ Network activities. We have established working relationships with other clinics in the local jurisdictions, the local health department, and other academic centers in Baltimore, to which we have expanded recruitment through the primary site. We anticipate being able to reach our long-term accrual targets.

### Conclusion

Engaging YHIV in ART care and treatment and ensuring treatment adherence and virologic suppression are necessary prerequisites in the attainment of better health-related quality of life and in alleviating the morbidity and mortality associated with HIV for YHIV. Well-designed and rigorously assessed youth-targeted adherence interventions that directly address the current disparities in treatment outcomes and continuum of care between youth and adults are essential. TECH2CHECK, if successful, will provide evidence for a strategy to improve adherence to care and viral suppression for YHIV, an often-marginalized population in need of tailored and well-resourced interventions that increase the likelihood of leading lives as near normal as possible. Preliminary recruitment and retention data support the feasibility and acceptability of the approach being used in the trial.

## Abbreviations

ACASI: audio, computer-assisted self-interview
ART: antiretroviral therapy
CHN: community health nurse
ML: monitoring labs
PfH: Partnership for Health
PLWH: persons living with HIV
RCT: randomized controlled trial
SMILE: Strategic Multisite Initiative for the Identification, Linkage, and Engagement in Care of Youth
SOC: standard of care
VL: viral load
YHIV: youth with HIV
